# The relation between synovitis of individual finger joints and grip force over the first 5 years in early rheumatoid arthritis — a cohort study

**DOI:** 10.1186/s13075-023-03212-6

**Published:** 2023-11-30

**Authors:** Maria Rydholm, Ankita Sharma, Lennart Jacobsson, Carl Turesson

**Affiliations:** 1 Rheumatology, Department of Clinical Sciences, Malmö, Lund University, Malmö, Sweden; 2 Department of Rheumatology, Skåne University Hospital, Malmö, Sweden; 3 Department of Rheumatology and Inflammation Research, Sahlgrenska Academy at Gothenburg University, Gothenburg, Sweden

**Keywords:** Rheumatoid arthritis, Finger joints, Hand strength, Synovitis

## Abstract

**Objective:**

The objective of this study was to investigate the relation between swelling and tenderness of individual finger joints and grip force in patients with early rheumatoid arthritis (RA).

**Methods:**

In an inception cohort of patients with early RA (symptom duration < 12 months), all patients were examined by the same rheumatologist, and grip force was measured using the Grippit instrument at inclusion, 1 and 5 years. The average grip force values of each hand were evaluated and expressed as % of expected values, based on age- and sex-specific reference values. Linear regression analyses were used to assess the cross-sectional relation between the involvement of individual finger joints and grip force. In generalized estimating equations, the impact of time-varying synovitis/tenderness on grip force over time was estimated. Analyses were adjusted for wrist involvement, erythrocyte sedimentation rate, and patient-reported pain.

**Results:**

In 215 patients with early RA, grip force was 39% of expected at diagnosis, and increased to 56% after 5 years. Synovitis of the first metacarpophalangeal (MCP) joint (60% and 69% at baseline in the right and left hand) was associated with reduced grip force at inclusion (adjusted ß − 9.2 percentage unit of expected grip force; 95% CI − 13.6 to − 4.8 for both hands combined) and at all follow-up evaluations. Synovitis of MCP I and MCP IV (12% at baseline) was significantly associated with reduced grip force over time in both hands. Proximal interphalangeal (PIP) joint swelling, and tenderness of MCP or PIP joints, had less impact on grip force.

**Conclusion:**

MCP I synovitis is the major contributor to reduced grip force in patients with early RA. This underlines the importance of the involvement of the thumb for impaired hand function in RA. MCP IV synovitis, but not PIP involvement or finger joint tenderness, also has a substantial impact on grip force.

**Supplementary Information:**

The online version contains supplementary material available at 10.1186/s13075-023-03212-6.

## Background

Rheumatoid arthritis (RA) often has a major impact on hand function. In severe cases, impairments due to successive progressive joint destruction generate disability and lead to limitations in performing activities of daily living (ADL) [1]. Joint involvement of the wrist and fingers, and related limitations in function and ADL, are common in both early and late RA [2, 3].

Patients with RA have been shown to have both reduced grip force in comparison with age- and sex-matched healthy populations [4–10], and reduced finger extension force [7, 10]. We have previously reported that although grip force improved over time in patients with early RA, most patients still had significantly reduced grip force 5 years after diagnosis [9].

Grip strength in the hands is dependent on many different components. General muscle strength, body weight, and overall physical functioning are independently associated with grip strength [11]. There are many basic components for the upper extremity (UE) which contribute to muscle strength in the hands. Different muscle groups of the distal UE cooperate in the production of the force, for example, the forearm flexor activation and the extensor synergist. Of particular importance are the extensor muscles of the wrist, whose task is to stabilize the structures of the wrist when the hand mobilizes grip force [12–14]. Wrist deformities are known to have a major impact on hand function [15–20].

Additionally, muscle strength in the upper extremity has been shown to correlate with grip force [20, 21]. Patients with RA had significantly impaired muscle strength in their shoulders 1.5 years after disease onset in comparison with age-matched subjects [8]. Already in early RA, the lean mass of arms and legs in both women and men was reduced compared to age-matched controls [22]. The shoulder, elbow, and wrist are the three joints in the upper extremity with the greatest contributions to the Health Assessment Questionnaire (HAQ) scores in patients with RA [23]. By contrast, there are limited data published on the relation between the involvement of specific joints in the hand and objective measures of hand function. Such findings could be useful for both targeted rehabilitation and other interventions in the health care of patients with RA. Their potential importance is underlined by the success of structured rehabilitation programs in RA [24–26]. For example, in the randomized controlled SARAH (Strengthening And stretching for Rheumatoid Arthritis of the Hand) trial, a tailored hand exercise program was shown to improve hand function in patients with RA [24].

Information on the contribution of individual joints to impaired grip strength may be helpful for guiding individualized management. We have previously reported that wrist synovitis and extensive metacarpophalangeal (MCP) joint synovitis are associated with reduced grip force in early RA, but the impact of individual finger joints has not been assessed [27]. The objective of this study was to investigate the relation between swelling and tenderness of individual finger joints and grip force in patients with early RA.

## Patients and methods

### Patients

An inception cohort of patients with early RA (symptom duration ≤ 12 months) recruited in 1995–2005, was investigated. The patients were diagnosed with RA by a rheumatologist and fulfilled the 1987 American College of Rheumatology (ACR) classification criteria for RA [28]. The study included individuals from a defined area, the city of Malmö, Sweden (population 260,000 in 2000). Patients were recruited from the rheumatology outpatient clinic of Malmö University Hospital, which was the only hospital serving the city, and from the four rheumatologists in private practice in Malmö. All patients gave their written informed consent to participate, and the study was approved by the Regional Ethical Review Board for southern Sweden (Lund, Sweden, LU 410–94, LU 311–02). The study was conducted according to the principles of the Helsinki Declaration.

### Clinical assessment

Patients were managed according to usual care, with no pre-specified protocol for pharmacotherapy or rehabilitation. In a structured follow-up program, all patients were examined by the same rheumatologist. Visits were scheduled at 6, 12, and 24 months as well as 5 years after inclusion. Using a standardized protocol, individual joints were assessed as swollen/not swollen and tender/not tender, and standard 28-joint swollen joint counts (SJC) and tender joint counts (TJC) were obtained. Disability was assessed using the Health Assessment Questionnaire Disability Index (HAQ-DI) [29], the Swedish validated version [30]. Patient-reported pain and patients’ global assessment of disease activity were assessed using visual analog scales (VAS; scale 0–100). Information on treatment was obtained as previously described [9]. Blood samples were obtained at the visit when the joint assessment was performed (within 1 h). C-reactive protein (CRP) and the erythrocyte sedimentation rate (ESR) were analyzed using standard methods at the Department of Clinical Chemistry, Malmö University Hospital.

### Assessment of grip force

Grip force (Newton, N) was measured by using the electronic instrument Grippit (AB Detektor, Gothenburg, Sweden). This was performed at the same visit as the joint assessment (within 1 h). All Grippit procedures were performed after 9.20 AM, in order to limit the impact of morning stiffness. The patient was seated comfortably in a chair without armrests, with the shoulder, arm, and hand in standard positions as previously described [31]. The other arm was resting on the table. Standardized instructions were given. When using this procedure, the test–retest scores for Grippit measures have been demonstrated to be high [31]. The grip force was measured alternately in the dominant hand and the non-dominant hand three times, and the mean of the three measurement values from each hand was used. Average values of the 10-s uninterrupted grip were obtained, as previously described [9]. Average grip force values of the right and left hand at inclusion and at the 1-year and 5-year follow-up visits were compared to the expected, based on age- and sex-specific reference values from a convenience sample from a cross-sectional study of volunteers in the region of Oslo, Norway [32]. Grip force values for each patient were expressed as % of the expected, based on the reference values.

### Statistics

Linear regression analyses were used to assess the cross-sectional relation between the involvement of individual finger proximal interphalangeal (PIP) and MCP joints and grip force in the corresponding hand at inclusion, 1 and 5 years. In the main analysis, synovitis and joint tenderness of individual joints in both hands were combined. As wrist synovitis, high ESR, and severe patient-reported pain (measured by VAS) were independently associated with reduced grip force in a previous study of this patient sample [27], and as we were interested in assessing the contribution of individual joints to impaired grip, the main analyses were adjusted for these covariates. By adjusting for ESR and pain, we corrected for potential confounding by general effects of inflammation on strength and impaired performance due to fear of pain. Unadjusted analyses, and analyses adjusted for only wrist involvement, were also performed. In addition, we investigated the relations between synovitis and joint tenderness and grip force in each hand in separate models.

Generalized estimating equations were used to assess the relation between finger joint synovitis and grip force over time. With repeated measures of grip force at inclusion, 1- and 5-year visits as the dependent variable, we investigated whether time-varying synovitis or tenderness of individual MCP and PIP joints were associated with reduced grip force over time, adjusted for time-varying wrist synovitis/tenderness, ESR and VAS pain. In addition, models adjusting for only wrist involvement and unadjusted models were constructed.

In exploratory analyses, the longitudinal analyses of the relation between individual synovitis/tenderness and grip force over time were stratified by autoantibody status (RF and/or anti-CCP2 positive vs seronegative for both antibodies).

## Results

### Patients

 A total of 215 patients with early RA (71% women; mean age 60 years) were investigated. The median symptom duration at inclusion was 7 months; interquartile range 5–10. Data on average grip force were available for 215 patients at inclusion, 216 patients at 1 year, and 175 patients at 5 years, although one patient was not able to perform the grip force test for the right hand at any of the visits (Table 1). Most patients were treated with Methotrexate (Table 1). At baseline, the mean average grip force was 102 N for the right hand, and 97 N for the left hand (mean 39% of expected for both hands). This increased over time to 137 N (mean 56% of expected) in the right hand and to 129 N (mean 55% of expected) in the left hand at 5 years (Table 1).

**Table 1:** Characteristics of the early RA cohort, at inclusion, 1 and 5 years of follow-up

**Characteristics of the early RA cohort†**	Inclusion^a^	**1 year** ^b^	**5 years** ^c^
Female sex *n* (%)	152 (71)	153 (71)	124 (71)
Age (years)	59.9 (14.7)	61 (14.6)	64.7 (14.3)
Symptom duration at inclusion (month); median (IQR)	7 (5–10)	7 (5‒10)	7 (5‒10)
RF positive % (*n*)	61 (132)	62 (135)	65 (114)
Anti-CCP positive % (*n*)	56 (104/187)	58 (108/186)	59 (90/153)
RF and/or anti-CCP positive % (*n*)	72 (154)	72 (155)	75 (132)
Swollen joints (0-28); median (IQR)	7 (5–11)	4 (2‒7)	4 (4‒7)
Tender joints (0-28); median (IQR)	4 (1–9)	2 (0‒5)	1 (1‒3)
DAS28 (0‒10)	4.64 (1.38)	3.65 (1.34)	3.59 (1.38)
HAQ (0‒3)	0.85 (0.62)	0.63 (0.59)	0.75 (0.66)
Patient’s global assessment (VAS 0‒100)	43 (26)	31 (24)	35 (24)
Pain (VAS 0‒100)	41 (27)	31 (24)	30 (23)
Average grip force right hand (N)	102 (76)	131 (85)	137 (93)
Average grip force right hand (% of expected)	39 (25)	51 (27)	56 (30)
Average grip force left hand (N)	97 (70)	120 (78)	130 (88)
Average grip force left hand (% of expected)	39 (25)	49 (27)	55 (30)
Methotrexate treatment % (*n*)	55 (119)	63 (137)	61 (106)
Other DMARD % (*n*)	30 (64)	31 (67)	25 (43)
Corticosteroid treatment % (*n*)	39 (83)	31 (66)	29 (51)
CRP (mg/l); median (IQR)	9 (< 9‒24)	< 9 (< 9‒10)	< 9 < 9‒10)
ESR (mm/h); median (IQR)	20 (10–40)	15 (7‒27)	16 (10‒25)

Values are mean (SD), unless otherwise indicated

*RA* Rheumatoid Arthritis, *IQR* Interquartile range, *RF* Rheumatoid factor, *anti-CCP* Anti-cyclic citrullinated peptide, *DAS28* Disease Activity Score in 28 joints, *HAQ* Health Assessment Questionnaire, *VAS* Visual analog scale, *N* Newton, *DMARD* Disease-modifying antirheumatic drug, *CRP* C-reactive protein, *ESR* Erythrocyte sedimentation rate

† All patients with > 1 grip force measure at inclusion and at 1 and 5 years

^a^*n* = 215

^b^*n* = 216

^c^*n* = 175

### Frequency of synovitis and tenderness involvement in early RA — over time

Among patients with data on average grip force at inclusion, 60% had synovitis of the MCP I joint in the right hand and 69% in the left hand. Synovitis in the MCP II joints was almost as common as in MCP I, whereas it was less frequently observed in MCP III and even less so in MCP IV and V (Table 2). PIP synovitis was observed less frequently than MCP synovitis. Proportions with current synovitis decreased somewhat over time in most joints, in particular in the right hand (Table 2). Tenderness was less common for MCP joints I–III for both hands and similar for MCP IV-V and all PIP joints, compared to swollen joints. The highest prevalence of tenderness at inclusion was seen in MCP I, 34% in the right hand and 38% in the left hand, which decreased to 20% at 5 years. There were similar changes over time for tenderness in other MCP and PIP joints.

**Table 2:** Synovitis and tenderness of individual joints at each follow-up visit in early RA patients

	Inclusion^a^		1-year follow-up^b^		**5-year follow-up**^c^	
	**Right**	**Left**	**Right**	**Left**	**Right**	**Left**
**Synovitis; ** ***n*** ** (%)**						
MCP I	130 (60)	149 (69)	100 (46)	131 (61)	83 (48)	108 (62)
MCP II	135 (63)	129 (60)	102 (48)	87 (40)	98 (56)	86 (49)
MCP III	102 (47)	95 (44)	62 (29)	59 (27)	59 (34)	57 (33)
MCP IV	26 (12)	27 (13)	9 (4)	3 (1)	9 (5)	9 (5)
MCP V	25 (12)	25 (12)	14 (6)	10 (5)	23 (13)	14 (8)
PIP I	35 (16)	42 (20)	17 (8)	20 (9)	10 (6)	10 (6)
PIP II	61 (28)	54 (25)	32 (15)	26 (12)	19 (11)	24 (14)
PIP III	73 (34)	70 (33)	36 (17)	36 (17)	31 (18)	26 (15)
PIP IV	44 (20)	56 (26)	22 (10)	34 (16)	16 (9)	23 (13)
PIP V	30 (14)	42 (20)	17 (8)	28 (13)	13 (8)	20 (11)
**Tenderness;** ***n*** **(%)**						
MCP I	73 (34)	82 (38)	46 (21)	56 (26)	34 (20)	35 (20)
MCP II	68 (32)	73 (34)	40 (19)	40 (18)	31 (18)	28 (16)
MCP III	60 (28)	62 (29)	29 (14)	30 (14)	23 (13)	24 (14)
MCP IV	36 (17)	32 (15)	17 (8)	14 (6)	14 (8)	11 (6)
MCP V	34 (16)	33 (15)	11 (5)	13 (6)	16 (9)	13 (7)
PIP I	39 (18)	35 (16)	20 (9)	19 (9)	14 (8)	14 (8)
PIP II	47 (22)	49 (23)	25 (12)	19 (9)	17 (10)	15 (9)
PIP III	51 (24)	55 (26)	25 (12)	32 (15)	21 (12)	14 (11)
PIP IV	53 (25)	48 (22)	15 (7)	23 (12)	14 (8)	17 (10)
PIP V	36 (17)	46 (21)	15 (7)	18 (8)	17 (10)	16 (9)

*MCP* Metacarpophalangeal joint, *PIP* Proximal interphalangeal joint

^a^Right *n* = 214. Left *n* = 215

^b^Right *n* = 215. Left *n* = 216

^c^Right *n* = 174. Left *n* = 175

### Relation between synovitis and tenderness of individual MCP and PIP joints and grip force in analyses of both hands combined

Synovitis of MCP I was associated with significantly reduced grip force at all visits (Table 3). There were similar associations for synovitis of the other MCP joints at inclusion, but not at later follow-ups (Table 3). Patients with tender MCP I joints had reduced grip force, with significant differences at inclusion and after 1 year (Table 3). Tenderness of other MCP and PIP joints was associated with lower grip force mainly at inclusion, with no statistically significant associations at the 5-year follow-up (Table 3).

**Table 3:** Relation between involvement of individual MCP and PIP joints and grip force, both hands combined (% of expected)

	**Inclusion** ^a^	**1-year follow-up** ^a^	**5-year follow-up** ^a^
Synovitis			
MCP I	** − 9.2 (− 13.6 to − 4.8)**	** − 7.5 (− 12.3 to − 2.7)**	** − 7.8 (− 13.6 to − 1.9)**
MCP II	** − 7.4 (− 11.7 to − 3.1)**	− 2.9 (− 7.8 to 2.0)	− 4.2 (− 10.2 to 1.9)
MCP III	** − 6.9 (− 11.0 to − 2.7)**	− 1.4 (− 6.9 to 4.2)	− 2.1 (− 8.5 to 4.2)
MCP IV	** − 8.7 (− 15.1 to − 2.4)**	− 9.1 (− 23.4 to 5.2)	− 3.5 (− 16.5 to 9.6)
MCP V	− 6.4 (− 13.0 to 0.3)	− 4.6 (− 14.9 to 5.7)	4.1 (− 5 − 7 to 13.9)
PIP I	0.6 (− 5.1 to 6.3)	− 3.9 (− 12.3 to 4.5)	− 6.9 (− 19.5 to 5.7)
PIP II	− 2.7 (− 7.5 to 2.0)	− 5.1 (− 12.1 to 1.8)	− 6.8 (− 15.6 to 2.1)
PIP III	** − 4.9 (− 9.4 to − 0.5)**	− 2.9 (− 9.2 to 3.5)	1.0 (− 6.9 to 9.0)
PIP IV	− 3.5 (− 8.5 to 1.5)	− 3.3 (− 10.2 to 3.6)	3.6 (− 5.6 to 12.8)
PIP V	− 3.7 (− 9.3 to 2.0)	− 5.0 (− 12.8 to 2.7)	− 1.8 (− 11.8 to 8.3)
Tender joints			
MCP I	** − 8.9 (− 13.4 to − 4.3)**	** − 5.9 (− 11.8 to − 0.1)**	− 7.2 (− 15.5 to 1.2)
MCP II	− 8.5 (− 13.0 to 4.0)	− 3.2 (− 9.7 to 3.2)	− 6.5 (− 15.2 to 2.2)
MCP III	** − 8.7 (− 13.9 to − 3.5)**	0.8 (− 6.8 to 8.4)	− 5.8 (− 15.1 to 3.6)
MCP IV	** − 12.1 (− 18.0 to − 6.3)**	− 7.5 (− 17.1 to 2.1)	− 6.7 (− 18.9 to 5.4)
MCP V	− ** − 7.6 (− 13.6 to − 1.6)**	− 9.0 (− 19.6 to 1.6)	5.8 (− 6.4 to 18.0)
PIP I	** − 5.8 (− 11.5 to − 0)**	− 6.9 (− 15.4 to 1.6)	− 5.9 (− 17.2 to 5.4)
PIP II	** − 8.0 (− 13.1 to** − **2.9)**	− 5.4 (− 13.6 to 2.8)	− 7.6 (− 18.4 to 3.2)
PIP III	** − 9.4 (− 14.3 to − 4.5)**	** − 8.4 (− 15.7 to − 1.1)**	− 4.0 (− 14.1 to 6.1)
PIP IV	** − 5.4 (− 10.4 to − 0.3)**	− 4.0 (− 12.6 to 4.6)	− 2.3 (− 13.2 to 8.6)
PIP V	** − 7.8 (− 13.2 to − 2.4)**	− 8.1 (− 17.3 to 1.1)	− 7.7 (− 18.1 to 2.8)

Linear regression analysis

^a^β (95% CI); adjusted for wrist synovitis/tenderness, erythrocyte sedimentation rate and pain (visual analog scale)

*MCP*, Metacarpophalangeal joint, *PIP* Proximal interphalangeal joint

In analyses adjusted for wrist synovitis/tenderness only, there were associations between MCP I involvement and reduced grip force at all time points (Supplementary Table 1). There were stronger relations for tenderness with reduced grip force in these models, and unadjusted analyses, where joint tenderness was consistently associated with lower grip force (Supplementary Table 2).

### Relation between synovitis and tenderness of individual MCP and PIP joints and grip force in separate, fully adjusted analyses of the right and left hand

In the right hand, synovitis of the first MCP joint was consistently associated with reduced grip force at all evaluations at inclusion, 1 and 5 years (Supplementary Table 3). Patients with MCP II and MCP III synovitis in the right hand also had reduced grip force at inclusion, but not after 1 and 5 years (Supplementary Table 3). PIP joint swelling (Table 3), and tenderness of MCP or PIP joints (Supplementary Table 4), had a lower impact on grip force. Results for the left hand showed that synovitis of the MCP joints I–III and V was consistently associated with reduced grip force at inclusion, but not at the 1- and 5-year follow-up (Supplementary Table 3). Tenderness in the right hand of the MCP joints I–IV and PIP III and V joint all had a significant impact on the grip strength at inclusion, but not at all at the 1 and 5-year follow-up (Supplementary Table 4). For the left hand, tenderness in MCP II and PIP III at inclusion had the greatest impact on grip strength, and tenderness of PIP III also had a significant impact at 1 year, but not at 5 years (Supplementary Table 4). Synovitis and tenderness in other joints were not associated with reduced grip strength at either time point (Supplementary Tables 3 and 4).

### Individual MCP/PIP involvement and grip force of the right and left hand — unadjusted analyses, and analyses adjusted for wrist involvement

In unadjusted models, and models adjusted for only wrist synovitis, there were also associations between MCP involvement and reduced grip force, in particular for MCP I in the right hand at all time points (Supplementary Table 5, Supplementary Table 6). Negative associations with joint tenderness were stronger in unadjusted analyses (Supplementary Table 7) and also to some extent in analyses adjusted for wrist involvement (Supplementary Table 8) compared to the fully adjusted models that also included ESR and patient-reported pain (Supplementary Table 6).

### MCP and PIP synovitis/tenderness and grip force over time

Synovitis of MCP I and MCP IV was significantly associated with reduced grip over time in both hands, with a similar finding for MCP V in the left hand only (Table 4). PIP synovitis had a lower impact on grip force over time (Table 4). Patients with tender MCP IV joints had lower grip force over time in both hands, whereas tenderness of MCP I and II was associated with reduced time-dependent grip force in the right hand only (Table 4). There were associations between joint tenderness and lower grip force over time in PIP I–III, but not consistently in both hands (Table 4).

**Table 4:** Relation over time between the involvement of individual MCP and PIP joint and grip force^a^

	**Right hand** ^b^	**Left hand** ^b^
**Synovitis**		
MCP I	** − 5.0 (− 8.3 to − 1.7)**	** − 3.7 (− 6.6 to − 0.8)**
MCP II	** − **1.7 (**− **4.9 to 1.5)	** − **1.7 (**− **4.8 to 1.4)
MCP III	** − **1.2 (**− **4.4 to 2.0)	** − **0.4 (**− **3.3 to 2.4)
MCP IV	** − 4.7 (− 9.3 to − 0.2)**	** − 6.1 (− 10.9 to − 1.4)**
MCP V	** − **3.0 (**− **7.7 to 1.7)	** − 6.0 (− 10.2 to − 1.8)**
PIP I	0.8 (**− **4.5 to 6.1)	** − **2.4 (**− **6.7 to 1.9)
PIP II	0.3 (**− **3.3 to 3.9)	** − **1.8 (**− **5.6 to 1.9)
PIP III	0.7 (**− **2.6 to 4.0)	** − **0.6 (**− **4.4 to 3.3)
PIP IV	** − **0.3 (**− **4.4 to 3.9)	0.1 (**− **4.2 to 4.3)
PIP V	** − **3.0 (**− **8.1 to 2.2)	1.1 (**− **4.6 to 6.9)
**Tenderness**		
MCP I	** − 5.8 (− 9.4 to − 2.2)**	** − **1.5 (**− **5.0 to 2.0)
MCP II	** − 4.0 (− 7.8 to − 0.2)**	** − **2.2 (**− **5.4 to 1.0)
MCP III	** − **2.9 (**− **7.1 to 1.2)	** − **0.3 (**− **3.8 to 3.2)
MCP IV	** − 9.0 (− 12.8 to − 5.2)**	** − 6.5 (− 10.8 to − 2.1)**
MCP V	** − **4.4 (**− **9.2 to 0.5)	** − **2.6 (**− **7.7 to 2.5)
PIP I	** − **1.7 (**− **6.9 to 3.5)	** − 4.9 (− 9.7 to − 0.2)**
PIP II	** − 4.5 (− 8.8 to − 0.2)**	** − **3.2 (**− **7.9 to 1.5)
PIP III	** − **4.3 (**− **8.7 to 0.1)	** − 3.9 (− 7.8 to − 0.1)**
PIP IV	** − **0.9 (**− **6.5 to 4.6)	** − **0.5 (**− **6.1 to 5.0)
PIP V	** − **4.0 (**− **8.9 to 0.8)	** − **1.9 (**− **7.5 to 3.8)

^a^(% of expected)

^b^β (95% CI), adjusted for wrist tenderness, erythrocyte sedimentation rate and pain (visual analog scale). Generalized estimating equations

*MCP* Metacarpophalangeal joint, *PIP* Proximal interphalangeal joint

In exploratory analyses stratified for autoantibody status (RF and/or anti-CCP2 positive vs seronegative for both antibodies), there was a significant association between MCP I synovitis and reduced grip force in both hands in seronegative patients (Supplementary Table 9), with similar trends in seropositive patients (Supplementary Table 10). Patients with synovitis or tenderness of MCP IV and V tended to have lower grip force in both subsets, although associations for MCP V involvement were stronger in seronegative patients (Supplementary Table 9, Supplementary Table 10).

## Discussion

In this study of patients with early RA, synovitis of the first MCP joint, which is a common disease feature, had a major impact on grip force, not only at diagnosis, but also later in the disease course. Swelling of other MCP joints, which for MCP III-V was less common compared to MCP I, was also associated with reduced grip force at inclusion, but not at other time points. These associations were significant in analyses adjusted for other factors known to affect the grip, i.e., wrist synovitis, systemic inflammation (measured by ESR), and patient-reported pain, indicating an independent effect on grip force by MCP synovitis. PIP synovitis appeared to affect grip force to a lesser extent. Exploratory analyses suggested similar patterns in seropositive and seronegative RA, although the estimates indicated stronger associations for MCP I and MCP V involvement with reduced grip force in seronegative RA.

Longitudinal analyses revealed lower grip force over time in those with synovitis of MCP I and MCP IV joints in both hands. Corresponding analyses of tender joints largely mirrored those of joint swelling, but with less consistent results in the right and the left hand. In analyses that were not adjusted for other factors known to influence grip force, including patient-reported pain, there were more and stronger associations between joint tenderness and reduced grip force, suggesting a general effect of pain/tenderness on performance when measuring grip force.

It is a well-known phenomenon that pain based on inflammation is triggered and worsened by motion and encumbrance in the affected joint [33]. Studies on patients with RA have demonstrated that treatment of MCP synovitis with local glucocorticoid injection has a major impact on hand function [34]. The results of our study are compatible with these findings, as we found that the MCP joints, the most affected finger joints, have the greatest effect on grip force.

Our results point to a particular importance of MCP I in this context. In general, the thumb is very important for hand function, as grip, pinch, and grasp are ineffective without thumb opposition [35]. Although the importance of MCP I involvement in RA has been recognized [36], quantitative data on its relation to grip force have been lacking.

Grip force is known to be affected by active joint inflammation [37], and has been used extensively for many years as an indicator of disease activity and disability [38–40]. It is considered a relevant measure of hand function in patients with RA, as power grip is the most commonly used functional grip [41].

Our findings that tenderness of individual MCP and PIP joints also had some impact on grip force are compatible with the study by Ozer et al. [42], who found that subclinical synovitis, detected by ultrasound, negatively affects the upper extremity function. The most commonly affected joints in that study were the right and left wrists, and the least involved joints were the left and right 3rd MCP and right and left 2nd PIP joints. The grip strength of the dominant hand was higher in patients with negative ultrasound power Doppler signal.

When measuring grip force, grasping an elliptical handle is preferred, as this measures force rather than pressure, contrary to squeezing a rubber bulb (e.g., Martin Vigorimeter) [41]. This procedure makes it easier to replicate the grip position of the hand in patients with deformities, stiffness, pain, etc. [41, 43], and it leads to strain on the whole hand and not only the thumb and index finger [43]. Electronic dynamometers are more sensitive, leading to better discrimination [41, 44] and lighter, making the instrument more manageable for those with a weak grip.

Similar to the Jamar dynamometer, Grippit uses test protocols with standardized position of the arm and hand, performed in accordance with the American Society of Hand Therapists recommendations [45]. To measure grip force three times per hand and to calculate the average of these measurements has been shown to give the highest reliability and validity in the evaluation of hand strength [45]. Another strength of the present study is that all the grip force measurements were performed using this standardized procedure. Furthermore, the same physician performed all the standardized joint examinations, using a structured protocol, within 1 h of the assessment of grip force.

There are several patient-related factors that may influence the result of grip force testing including age and gender [46], hand dominance [47] number of digits used for the grip [48], and the time of the day for testing [49], In the present study, measured grip force values were compared to the expected, based on age and sex-specific reference values. We consistently measured grip force using an electronic dynamometer with an elliptical handle at a standard time of the day.

Limitations of this study are related to the fact that patients were included before the principle of treat to target management of RA was established. Patients diagnosed in more recent years may experience less joint involvement and better hand function over time. However, associations at diagnosis should not be affected by changes in therapy standards. Although we adjusted for several relevant factors, including patient-reported pain, we did not have access to data on localized pain or hand discomfort. Furthermore, data on psychologic factors that may affect patient performance in functional tests were not collected in the present study.

To sum up, we found that synovitis of MCP I, which occurs in the majority of patients with early RA, had the strongest association with grip strength. This information could be helpful for individualizing therapy, including intra-articular glucocorticoid injections, systemic anti-rheumatic treatment, and structured hand training. Such individualized treatment and hand rehabilitation may contribute to improved ability to perform daily activities, in particular those requiring substantial grip strength, and hence to improved health and quality of life for patients with RA.

## Conclusion

- MCP synovitis of the thumb is common in patients with early RA, and a major contributor to reduced grip force over the first 5 years.
- Although less frequent, the involvement of MCP IV and MCP V may also have a significant impact on hand function.
- Despite improvement, grip force was still substantially reduced compared to the general population 5 years after RA diagnosis.
- This underlines the importance of both efficient treatment of synovitis and structured hand training in early RA.

### Supplementary Information

**Additional file 1: Supplementary Table 1. **Relation between involvement of individual MCP and PIP joints and grip force (% of expected). Adjusted for wrist synovitis/tenderness. **Supplementary Table 2.** Relation between involvement of individual MCP and PIP joints and grip force (% of expected). Unadjusted. **Supplementary Table 3.** Relation between synovitis of individual MCP and PIP joints and grip force (% of expected), separate for the right and left hand, fully adjusted. **Supplementary Table 4.** Relation between tenderness of individual MCP and PIP joints and grip force (% of expected), separate for the right and left hand, fully adjusted. **Supplementary Table 5.** Relation between synovitis of individual MCP and PIP joints and grip force of the right hand or left hand (% of expected) in patients with early RA. Unadjusted. **Supplementary Table 6.** Relation between synovitis of individual MCP and PIP joints and grip force of the right hand or left hand (% of expected) in patients with early RA. Adjusted for wrist synovitis. **Supplementary Table 7.** Relation between joint tenderness of individual MCP and PIP joints and grip force of the right hand or left hand (% of expected) in patients with early RA. Unadjusted. **Supplementary Table 8.** Relation between tenderness of individual MCP and PIP joints and grip force of the right hand or left hand (% of expected) in patients with early RA. Adjusted for wrist tenderness. **Supplementary Table 9.** RF/anti-CCP positive patients with RA. Relation over time between involvement of individual MCP and PIP joints and grip force. **Supplementary Table 10.** RF- and anti-CCP negative patients with RA. Relation over time between involvement of individual MCP and PIP joints and grip force.

## Data Availability

The datasets used and/or analyzed during the current study are available from the corresponding author upon reasonable request.

## Abbreviations

RA: Rheumatoid arthritis
MCP: Metacarpophalangeal
PIP: Proximal interphalangeal
ADL: Activities of daily living
UE: Upper extremity
HAQ: Health Assessment Questionnaire
SARAH: Strengthening And stretching for Rheumatoid Arthritis of the Hand
ACR: American College of Rheumatology
SJC: Swollen joint counts
TJC: Tender joint counts
HAQ-DI: Health Assessment Questionnaire Disability Index
VAS: Visual analog scales
CRP: C-reactive protein
ESR: Erythrocyte sedimentation rate
