# Supplementary material for: The relation between synovitis of individual finger joints and grip force over the first 5 years in early rheumatoid arthritis — a cohort study
Source: Arthritis Res Ther. 2023 Nov 30;25:231. doi: 10.1186/s13075-023-03212-6 (PMC10688125; doi:10.1186/s13075-023-03212-6)
Supplement: Supplementary file 1 — Additional file 1: Supplementary Table 1. Relation between involvement of individual MCP and PIP joints and grip force (% of expected). Adjusted for wrist synovitis/tenderness. Supplementary Table 2. Relation between involvement of individual MCP and PIP joints and grip force (% of expected). Unadjusted. Supplementary Table 3. Relation between synovitis of individual MCP and PIP joints and grip force (% of expected), separate for the right and left hand, fully adjusted. Supplementary Table 4. Relation between tenderness of individual MCP and PIP joints and grip force (% of expected), separate for the right and left hand, fully adjusted. Supplementary Table 5. Relation between synovitis of individual MCP and PIP joints and grip force of the right hand or left hand (% of expected) in patients with early RA. Unadjusted. Supplementary Table 6. Relation between synovitis of individual MCP and PIP joints and grip force of the right hand or left hand (% of expected) in patients with early RA. Adjusted for wrist synovitis. Supplementary Table 7. Relation between joint tenderness of individual MCP and PIP joints and grip force of the right hand or left hand (% of expected) in patients with early RA. Unadjusted. Supplementary Table 8. Relation between tenderness of individual MCP and PIP joints and grip force of the right hand or left hand (% of expected) in patients with early RA. Adjusted for wrist tenderness. Supplementary Table 9. RF/anti-CCP positive patients with RA. Relation over time between involvement of individual MCP and PIP joints and grip force. Supplementary Table 10. RF- and anti-CCP negative patients with RA. Relation over time between involvement of individual MCP and PIP joints and grip force. [file 13075_2023_3212_MOESM1_ESM.docx]

| Supplementary Table 1. Relation between involvement of individual MCP and PIP joints and grip force (% of expected).  Adjusted for wrist synovitis/tenderness | | | |
| --- | --- | --- | --- |
|  | **Inclusion*** | **1-year follow-up*** | **5-years follow-up*** |
| Synovitis | | | |
| MCP I | **-11.2 (-16.0 to -6.4)** | **-8.4 (-13.4 to -3.3)** | **-8.7 (-14.9 to -2.6)** |
| MCP II | **-7.2 (-11.9 to -2.5)** | -4.6 (-9.8 to 0.5) | **-9.1 (-15.2 to -3.0)** |
| MCP III | **-6.3 (-10.8 to -1.7)** | -5.1 (-10.9 to 0.7) | **-7.2 (-13.6 to -0.8)** |
| MCP IV | **-10.8 (-17.6 to -3.9)** | -10.1 (-25.5 to 5.3) | -4.7 (-18.6 to 9.2) |
| MCP V | **-10.5 (-17.6 to -3.4)** | -4.2 (-15.3 to 7.0) | -2.2 (-12.3 to 7.9) |
| PIP I | -3.5 (-9.6 to 2.5) | -6.1 (-15.1 to 2.9) | -12.1 (-25.3 to 1.1) |
| PIP II | **-5.5 (-10.6 to -0.4)** | **-7.5 (-14.9 to -0.2)** | **-12.4 (-21.5 to -3.2)** |
| PIP III | **-6.2 (-11.0 to -1.4)** | -3.9 (-10.6 to 2.9) | -2.0 (-10.3 to 6.4) |
| PIP IV | -3.0 (-8.4 to 2.4) | -3.8 (-11.3 to 3.6) | -1.1 (-10.8 to 8.5) |
| PIP V | -4.3 (-10.4 to 1.8) | -6.4 (-14.7 to 1.9) | -8.2 (-18.5 to 2.1) |
| Tender joints | | | |
| MCP I | **-11.5 (-16.4 to -6.6)** | **-8.4 (-14.6 to -2.2)** | **-10.7 (-19.4 to -2.1)** |
| MCP II | **-11.2 (-16.1 to -6.3)** | -5.7 (-12.5 to 1.0) | **-11.2 (-20.1 to -2.2)** |
| MCP III | **-8.7 (-13.9 to -3.5)** | -4.7 (-12.5 to 3.2) | **-11.2 (-20.8 to -1.5)** |
| MCP IV | **-16.1 (-22.3 to -9.9)** | -8.9 (-19.1 to 1.3) | -9.8 (-22.6 to 3.0) |
| MCP V | **-11.3 (-17.7 to -5.0)** | **-12.4 (-23.7 to -1.2)** | 0.7 (-12.1 to 13.5) |
| PIP I | **-9.8 (-15.9 to -3.6)** | **-9.7 (-18.7 to -0.8)** | **-12.6 (-24.3 to -1.0)** |
| PIP II | **-11.4 (-16.9 to -5.9)** | -8.6 (-17.1 to 0) | **-12.8 (-24.0 to -1.6)** |
| PIP III | **-12.4 (-17.7 to -7.2)** | **-8.9 (-16.5 to -1.3)** | -8.7 (-19.0 to 1.7) |
| PIP IV | **-8.6 (-14.0 to -3.1)** | -5.8 (-15.0 to 3.3) | -6.3 (-17.8 to 5.1) |
| PIP V | **-10.3 (-16.1 to -4.4)** | **-13.2 (-22.9 to -3.6)** | **-13.2 (-24.1 to -2.3)** |
| Linear regression analysis *β (95 % CI).  MCP = Metacarpophalangeal joint; PIP = Proximal interphalangeal joint. | | | |

| Supplementary Table 2. Relation between involvement of individual MCP and PIP joints  and grip force (% of expected)  Unadjusted. | | | | |
| --- | --- | --- | --- | --- |
|  | **Inclusion*** | **1-year follow-up*** | **5-years follow-up*** | |
| Synovitis | | | | |
| MCP I | **-14.2 (-19.0 to -9.4)** | **-10.5 (-15.6 to -5.4)** | | **-12.3 (-18.5 to -6.2)** |
| MCP II | **-9.7 (-14.5 to -4.9)** | **-7.4 (-12.5 to -2.2)** | | **-12.6 (-18.8 to -6.5)** |
| MCP III | **-8.2 (-12.9 to -3.5)** | **-8.7 (-14.3 to -3.0)** | | **-10.3 (-16.9 to -3.8)** |
| MCP IV | **-14.0 (-21.1 to -6.9)** | **-16.2 (-31.7 to -0.7)** | | -13.7 (-27.7 to 0.4) |
| MCP V | **-14.2 (-21.4 to -6.9)** | -9.4 (-20.6 to 1.8) | | -9.7 (-19.8 to 0.4) |
| PIP I | **-6.9 (-13.1 to -0.8)** | **-9.4 (-18.5 to -0.2)** | | **-20.1 (-33.4 to -6.9)** |
| PIP II | **-6.2 (-11.5 to -0.8)** | **-9.8 (-17.3 to -2.3)** | | **-16.7 (-26.1 to -7.4)** |
| PIP III | **-7.5 (-12.5 to -2.6)** | -5.8 (-12.7 to 1.1) | | -7.8 (-16.2 to 0.6) |
| PIP IV | -5.0 (-10.6 to 0.6) | -4.6 (-12.2 to 3.0) | | -5.9 (-15.8 to 4.0) |
| PIP V | -5.1 (-11.5 to 1.3) | -10.0 (-18.4 to -1.7) | | **-12.3 (-22.9 to -1.7)** |
| Tender joints | | | | |
| MCP I | **-14.8 (-19.5 to -10.1)** | **-12.9 (-18.8 to -7.0)** | | **-17.3 (-24.9 to -9.7)** |
| MCP II | **-14.1 (-19.0 to -9.3)** | **-10.7 (-17.2 to -4.2)** | | **-17.7 (-25.9 to -9.6)** |
| MCP III | **-11.9 (-17.1 to -6.8)** | **-11.6 (-19.0 to -4.2)** | | **-18.1 (-27.1 to -9.2)** |
| MCP IV | **-17.8 (-24.1 to -11.6)** | **-16.0 (-25.9 to -6.2)** | | **-19.9 (-31.8 to -7.9)** |
| MCP V | **-13.1 (-19.5 to -6.7)** | **-17.7 (-28.8 to -6.7)** | | **-13.3 (-24.5 to -2.1)** |
| PIP I | **-13.0 (-19.2 to -6.9)** | **-14.7 (-23.5 to -5.8)** | | **-19.9 (-31.2 to -8.6)** |
| PIP II | **-13.2 (-18.7 to -7.6)** | **-13.9 (-22.3 to -5.5)** | | **-20.5 (-31.1 to -9.9)** |
| PIP III | **-14.6 (-19.9 to -9.3)** | **-13.2 (-20.7 to -5.8)** | | **-16.7 (-26.3 to -7.0)** |
| PIP IV | **-10.1 (-15.6 to -4.6)** | **-11.3 (-20.3 to -2.3)** | | **-15.2 (-26.0 to -4.3)** |
| PIP V | **-12.0 (-17.9 to -6.1)** | **-18.1 (-27.6 to -8.6)** | | **-20.1 (-30.5 to -9.6)** |
| Linear regression analysis, *β (95 % CI); MCP = Metacarpophalangeal joint; PIP = Proximal interphalangeal joint. | | | | |

| Supplementary Table 3. Relation between synovitis of individual MCP and PIP joints and grip force (% of expected) | | | | | | |
| --- | --- | --- | --- | --- | --- | --- |
|  | **Inclusion*** | | | **1-year follow-up*** | | **5-years follow-up*** |
| Right hand | | | | | | |
| MCP I | | **-10.8 (-17.1 to-4.4)** | **-8.0 (-14.8 to -1.2)** | | **-8.2 (-16.4 to 0)** | |
| MCP II | | **-7.5 (-13.9 to -1.1)** | -0.6 (-7.6 to 6.4) | | -0.6 (-8.2 to 9.5) | |
| MCP III | | **-7.4 (-13.5 to -1.3)** | -1.3 (-9.4 to 6.8) | | -4.5 (-13.4 to 4.5) | |
| MCP IV | | -8.9 (-18.5 to 0.7) | -8.8 (-25.7 to 8.1) | | -4.0 (-22.1 to 14.0) | |
| MCP V | | -2.5 (-12.6 to 7.6) | -2.6 (-16.5 to 11.2) | | 7.4 (-5.2 to 20.0) | |
| PIP I | | 2.0 (-6.4 to 10.9) | -1.6 (-14.0 to 10.7) | | -5.1 (-23.3 to 13.2) | |
| PIP II | | -0.8 (-7.7 to 6.2) | -3.9 (-13.3 to 5.5) | | -3.2 (-16.2 to 9.8) | |
| PIP III | | -5.6 (-12.1 to 1.0) | 2.5 (-6.5 to 11.6) | | 1.9 (-9.0 to 12.9) | |
| PIP IV | | -4.1 (-11.8 to 3.6) | -1.9 (-12.8 to 9.0) | | -0.1 (-14.2 to 14.1) | |
| PIP V | | -7.2 (-16.0 to 1.7) | -10.3 (-22.8 to 2.3) | | -5.6 (-21.2 to 9.9) | |
| Left hand | | | | | | |
| MCP I | | **-7.5 (-13.8 to -1.1)** | -6.4 (-13.4 to 0.6) | | -7.8 (-16.6 to 1.0) | |
| MCP II | | **-7.5 (-13.3 to -1.6)** | -5.7 (-12.6 to 1.3) | | -8.1 (-16.7 to 0.5) | |
| MCP III | | **-6.1 (-11.9 to -0.2)** | -1.7 (-9.5 to 6.1) | | 0.3 (-8.9 to 9.4) | |
| MCP IV | | -8.2 (-16.8 to 0.4) | -14.1 (-42.5 to 14.3) | | -3.0 (-22.5 to 16.5) | |
| MCP V | | **-9.7 (-18.6 to -0.8)** | -8.2 (-24.1 to 7.7) | | -1.1 (-17.0 to 14.8) | |
| PIP I | | -0.6 (-8.3 to 7.0) | -5.6 (-17.2 to 6.0) | | -8.8 (-26.6 to 9.1) | |
| PIP II | | -4.7 (-11.4 to 1.7) | -6.4 (-16.9 to 4.0) | | -10.1 (-22.4 to 2.3) | |
| PIP III | | -4.4 (-10.5 to 1.7) | -8.0 (-17.0 to 1.0) | | 0.4 (-11.5 to 12.2) | |
| PIP IV | | -3.0 (-9.7 to 3.6) | -3.8 (-12.9 to 5.3) | | 6.7 (-5.6 to 19.1) | |
| PIP V | | -0.7 (-8.1 to 6.7) | -1.0 (-11.0 to 9.1) | | 0.9 (-12.6 to 14.4) | |
| Linear regression analysis  *β (95 % CI), adjusted for wrist tenderness, erythrocyte sedimentation rate and pain (visual analogue scale); MCP = Metacarpophalangeal joint; PIP = Proximal interphalangeal joint. | | | | | | |

| Supplementary Table 4. Relation between tenderness of individual MCP and PIP joints and grip force (% of expected) | | | | |
| --- | --- | --- | --- | --- |
|  | **Inclusion*** | **1-year follow-up*** | | **5-years follow-up*** |
| Right hand | | | | |
| MCP I | **-13.3 (-20.0 to -6.6)** | | -7.7 (-16.2 to 0.8) | -4.8 (-16.8 to 7.3) |
| MCP II | **-10.2 (-17.0 to -3.4)** | | -2.3 (-11.4 to 6.8) | -1.7 (-14.1 to 10.7 |
| MCP III | **-7.7 (-14.8 to -0.5)** | | 2.3 (-8.4 to 13.1) | -0.1 (-13.9 to 13.6) |
| MCP IV | **-13.6 (-22.2 to -5.0)** | | -10.2 (-23.4 to 3.1) | -3.7 (-19.4 to 12.1) |
| MCP V | -7.1 (-16.2 to 2.1) | | -13.0 (-28.8 to 2.9) | 9.7 (-7.5 to 26.9) |
| PIP I | -6.4 (-14.7 to 1.9) | | -6.5 (-18.6 to 5.6) | -1.0 (-17.1 to 15.0) |
| PIP II | -7.7 (-15.4 to 0.1 | | -7.2 (-18.3 to 3.8) | -8.0 (-22.5 to 6.5) |
| PIP III | **-9.6 (-17.0 to -2.2)** | | -5.9 (-16.9 to 5.1) | -1.5 (-15.3 to 12.4) |
| PIP IV | -3.8 (-11.2 to 3.5) | | -7.1 (-20.8 to 6.7) | -4.5 (-20.3 to 11.3) |
| PIP V | **-9.2 (-17.7 to -0.8)** | | -11.4 (-25.6 to 2.8) | -5.3 (-19.8 to 9.3) |
| Left hand | | | | |
| MCP I | -4.8 (-11.3 to 1.6) | | -4.4 (-12.7 to 4.0) | -9.7 (-21.5 to 2.1) |
| MCP II | **-6.6 (-13.2 to -0.1)** | | -4.2 (-13.6 to 5.1) | -10.6 (-22.9 to 1.7) |
| MCP III | -3.4 (-10.3 to 3.6) | | -0.7 (-11.7 to 10.2) | -10.0 (-23.0 to 3.1) |
| MCP IV | -8.0 (-16.4 to 0.3) | | -4.8 (-19.0 to 9.5) | -12.4 (-32.0 to 7.3) |
| MCP V | -4.2 (-12.5 to 4.2) | | -5.8 (-20.4 to 8.8) | 3.7 (-14.1 to 21.5) |
| PIP I | -5.7 (-13.7 to 2.4) | | -7.4 (-19.5 to 4.8) | -10.8 (-26.9 to 5.4) |
| PIP II | -6.4 (-13.4 to 0.6) | | -3.5 (-15.9 to 9.0) | -7.2 (-23.7 to 9.3) |
| PIP III | **-8.2 (-14.9 to -1.4)** | | **-10.2 (-20.1 to -0.3)** | -6.6 (-21.5 to 8.4) |
| PIP IV | -5.0 (-12.1 to 2.1) | | -1.8 (-13.0 to 9.5) | -0.1 (-15.8 to 15.7) |
| PIP V | -5.6 (-12.6 to 1.5) | | -5.5 (-17.9 to 6.9) | -9.8 (-25.1 to 5.5) |
| Linear regression analysis  *β (95 % CI), adjusted for wrist tenderness, erythrocyte sedimentation rate and pain (visual analogue scale); MCP = Metacarpophalangeal joint; PIP = Proximal interphalangeal joint. | | | | |

| Supplementary Table 5. Relation between synovitis of individual MCP and PIP joints and grip force of the right hand or left hand (% of expected) in patients with early RA.  Unadjusted | | | | | | |
| --- | --- | --- | --- | --- | --- | --- |
|  | **Inclusion*** | | | **1-year follow-up*** | | **5-years follow-up*** |
| Right hand | | | | | | |
| MCP I | | **-15.4 (-22.1 to-8.7)** | | **-11.9 (-19.0 to -4.7)** | **-12.7 (-21.4 to -4.0)** | |
| MCP II | | **-10.3 (-17.3 to -3.4)** | | -5.0 (-12.4 to 2.2) | **-9.7 (-18.6 to -0.9)** | |
| MCP III | | **-8.6 (-15.3 to -1.8)** | | **-10.6 (-18.5 to -2.7)** | **-13.2 (-22.5 to -4.0)** | |
| MCP IV | | **-13.9 (-24.2 to -3.6)** | | -16.9 (-34.9 to 1.1) | -13.8 (-33.8 to 6.2) | |
| MCP V | | **-10.7 (-21.2 to -0.2)** | | -7.1 (-21.8 to 7.6) | 7.2 (-20.3 to 6.0) | |
| PIP I | | -6.8 (-16.0 to 2.3) | | -6.0 (-19.5 to 7.4) | **-21.8 (-40.6 to -2.9)** | |
| PIP II | | -6.0 (-13.6 to 1.4) | | -6.8 (-17.0 to 3.4) | -13.0 (-27.2 to 1.2) | |
| PIP III | | **-9.9 (-17.0 to -2.9)** | | -1.0 (-10.8 to 8.7) | -8.7 (-20.2 to 2.9) | |
| PIP IV | | -4.0 (-12.4 to 4.4) | | -4.3 (-16.3 to 7.7) | -12.0 (-27.3 to 3.3) | |
| PIP V | | **-9.2 (-19.0 to -0.6)** | | **-19.0 (-32.2 to -5.8)** | -14.5 (-31.4 to 2.3) | |
| Left hand | | | | | | |
| MCP I | | **-12.4 (-19.4 to -5.4)** | | **-9.0 (-16.4 to -1.5)** | **-12.3 (-21.3 to -3.4)** | |
| MCP II | | **-7.9 (-14.5 to -1.2)** | | **-9.9 (-17.3 to -2.6)** | **-15.7 (-24.2 to -7.1)** | |
| MCP III | | **-7.6 (-14.2 to -1.0)** | | -6.7 (-14.9 to 1.4) | -7.4 (-16.8 to 2.0) | |
| MCP IV | | **-14.3 (-24.1 to -4.5)** | | -16.4 (-47.5 to 14.8) | -13.5 (-33.5 to 6.5) | |
| MCP V | | **-17.6 (-24.7 to -7.6)** | | -12.9 (-30.2 to 4.4) | -13.9 (-30.1 to 2.4) | |
| PIP I | | **-9.0 (-17.3 to -0.7)** | | -12.1 (-24.6 to 0.4) | -18.5 (-37.4 to 0.4) | |
| PIP II | | -6.8 (-14.4 to 0.7) | **-13.6 (-24.7 to -2.6)** | | **-19.8 (-32.4 to -7.2)** | |
| PIP III | | -5.4 (-12.4 to 1.6) | **-10.6 (-20.3 to -0.9)** | | -6.9 (-19.4 to 5.5) | |
| PIP IV | | -6.2 (-13.8 to 1.2) | -4.5 (-14.5 to 5.6) | | -1.4 (-14.5 to 11.7) | |
| PIP V | | 1.5 (-6.7 to 9.0) | -4.0 (-14.9 to 6.9) | | -10.8 (-24.7 to 3.0) | |
| **β (95% confidence interval). MCP = Metacarpophalangeal joint; PIP = Proximal interphalangeal joint. | | | | | | |

| Supplementary Table 6. Relation between synovitis of individual MCP and PIP joints and grip force of the right hand or left hand (% of expected) in patients with early RA. Adjusted for wrist synovitis | | | | |
| --- | --- | --- | --- | --- |
|  | **Inclusion*** | **1-year follow-up*** | | **5-years follow-up*** |
| Right hand | | | | |
| MCP I | **-13.2 (-20.0 to -6.5)** | | **-10.1 (-17.3 to -2.9)** | **-8.8 (-17.3 to -0.2)** |
| MCP II | **-8.8 (-15.7 to -1.9)** | | -2.3 (-9.8 to 5.2) | -3.2 (-12.2 to 5.8) |
| MCP III | **-8.0 (-14.5 to -1.4)** | | -7.2 (-15.6 to 1.3) | -8.9 (-18.0 to 0.1) |
| MCP IV | **-11.7 (-22.0 to -1.4)** | | -11.7 (-29.8 to 6.5) | -6.3 (-25.6 to 12.9) |
| MCP V | -7.9 (-18.4 to 2.7) | | -1.7 (-16.7 to 13.2) | 0.9 (-12.0 to 13.8) |
| PIP I | -1.4 (-10.5 to 7.7) | | -3.1 (16.4 to 10.3) | -9.7 (-28.6 to 9.2) |
| PIP II | -4.2 (-11.6 to 3.1) | | -5.9 (-15.9 to 4.1) | -8.4 (-22.0 to 5.2) |
| PIP III | -8.3 (-15.2 to -1.3) | | 0.4 (-9.2 to 10.0) | -0.5 (-12.1 to 11.1) |
| PIP IV | -2.2 (-10.4 to 6.1) | | -3.1 (-14.9 to 8.8) | -5.2 (-20.1 to 9.6) |
| PIP V | -8.8 (-18.3 to 0.7) | | **-15.3 (-28.7 to -1.9)** | -9.8 (-25.9 to 6.3) |
| Left hand | | | | |
| MCP I | **-8.9 (-15.8 to -2.0)** | | -6.0 (-13.4 to 1.4) | -9.0 (-18.1 to 0.2) |
| MCP II | -5.8 (-12.2 to 0.6) | | **-7.4 (-14.7 to -0.1)** | **-14.1 (-22.5 to -5.7)** |
| MCP III | -4.3 (-10.7 to 2.1) | | -3.5 (-11.6 to 4.6) | -5.3 (-14.6 to 4.0) |
| MCP IV | **-9.5 (-18.9 to -0.2)** | | -11.0 (-41.2 to 19.3) | -3.9 (-24.4 to 16.6) |
| MCP V | **-12.8 (-22.4 to -3.2)** | | -8.5 (-25.4 to 8.4) | -7.1 (-23.6 to 9.4) |
| PIP I | -5.2 (-13.3 to 2.8) | | -8.3 (-20.6 to 4.0) | -13.7 (-32.5 to 5.0) |
| PIP II | -7.0 (-14.1 to 0.1) | | -9.5 (-20.5 to 1.6) | -16.0 (-28.6 to -3.3) |
| PIP III | -4.2 (-10.9 to 2.4) | | -7.9 (-17.4 to 1.6) | -3.2 (-15.5 to 9.2) |
| PIP IV | -3.6 (-10.8 to 3.6) | | -3.8 (-13.5 to 5.9) | 2.1 (-10.9 to 15.0) |
| PIP V | -0.5 (-8.5 to 7.6) | | -0.1 (-10.8 to 10.6) | -7.3 (-21.0 to 6.4) |
| *β (95% confidence interval). MCP = Metacarpophalangeal joint; PIP = Proximal interphalangeal joint. | | | | |

| Supplementary Table 7. Relation between joint tenderness of individual MCP and PIP joints and grip force of the right hand or left hand (% of expected) in patients with early RA.  Unadjusted. | | | | | |
| --- | --- | --- | --- | --- | --- |
|  | **Inclusion*** | | **1-year follow-up*** | | **5-years follow-up*** |
| Right hand | | | | | |
| MCP I | | **-18.0 (-24.8 to -11.3)** | **-15.0 (-23.7 to -6.4)** | **-18.7 (-29.6 to -7.9)** | |
| MCP II | | **-15.6 (-22.6 to -8.5)** | **-9.7 (-19.0 to -0.4)** | **-16.7 (-28.0 to -5.3)** | |
| MCP III | | **-12.5 (-19.9 to -5.1)** | **-10.7 (-21.3 to -0.2)** | **-17.4 (-30.3 to -4.4)** | |
| MCP IV | | **-20.1 (-28.8 to -11.4)** | **-17.6 (-30.8 to -4.3)** | **-18.2 (-34.3 to -2.0)** | |
| MCP V | | **-14.6 (-23.7 to -5.5)** | **-20.5 (-36.8 to -4.2)** | **-15.3 (-30.6 to -0.1)** | |
| PIP I | | **-12.0 (-20.7 to -3.3)** | **-14.4 (-26.8 to -2.0)** | **-18.5 (-34.6 to -2.3)** | |
| PIP II | | **-13.7 (-21.8 to -5.6)** | **-14.4 (-25.6 to -3.2)** | **-20.9 (-35.6 to -6.2)** | |
| PIP III | | **-13.9 (-21.7 to -6.1)** | **-11.6 (-22.8 to -0.3)** | **-16.6 (-30.0 to -3.1)** | |
| PIP IV | | **-8.1 (-15.9 to -0.2)** | **-16.8 (-30.9 to -2.7)** | **-18.4 (-34.6 to -2.3)** | |
| PIP V | | **-13.4 (-22.3 to -4.4)** | **-27.1 (-40.9 to -13.2)** | **-19.6 (-34.4 to -4.9)** | |
| Left hand | | | | | |
| MCP I | | **-11.7 (-18.3 to -5.0)** | **-10.9 (-19.1 to -2.7)** | **-15.9 (-26.7 to -5.1)** | |
| MCP II | | **-12.7 (-19.5 to -5.9)** | **-11.7 (-21.0 to -2.5)** | **-19.0 (-30.7 to -7.2)** | |
| MCP III | | **-11.3 (-18.5 to -4.1)** | **-12.5 (-22.9 to -2.0)** | **-18.8 (-31.4 to -6.3)** | |
| MCP IV | | **-15.3 (-24.4 to -6.2)** | -14.4 (-29.2 to 0.3) | **-22.1 (-40.1 to -4.2)** | |
| MCP V | | **-11.4 (-20.5 to -2.3)** | -15.2 (-30.5 to 0) | -10.9 (-22.7 to 5.9) | |
| PIP I | | **-14.0 (-22.8 to -5.2)** | **-15.0 (-27.7 to -2.2)** | **-21.4 (-37.4 to -5.4)** | |
| PIP II | | **-12.6 (-20.3 to -4.9)** | **-13.7 (-26.4 to -0.9)** | **-20.1 (-35.7 to -4.6)** | |
| PIP III | | **-15.3 (-22.6 to -7.9)** | **-14.4 (-24.5 to -4.3)** | **-16.8 (-30.9 to -2.8)** | |
| PIP IV | | **-12.2 (-21.0 to -4.4)** | -7.3 (-19.1 to 4.5) | -12.4 (-27.3 to 2.4) | |
| PIP V | | **-10.9 (-18.8 to -2.9)** | **-28.0 (-41.9 to -14.1)** | **-20.6 (-35.6 to -5.5)** | |
| *β (95% confidence interval). Values are % (n) unless otherwise indicated. MCP = Metacarpophalangeal joint; PIP = Proximal interphalangeal joint. | | | | | |

| Supplementary Table 8. Relation between tenderness of individual MCP and PIP joints and grip force of the right hand or left hand (% of expected) in patients with early RA  Adjusted for wrist tenderness | | | | | | | |  |
| --- | --- | --- | --- | --- | --- | --- | --- | --- |
|  | **Inclusion*** | | **1-year follow-up*** | | | **5-years follow-up*** | |  |
| Right hand | | | | | | | |  |
| MCP I | **-15.5 (-22.5 to -8.4)** | | | **-10.8 (-19.7 to -1.9)** | | -8.6 (-21.0 to 3.7) | |  |
| MCP II | **-13.0 (-20.2 to -5.8)** | | | -4.5 (-14.0 to 5.1) | | -5.3 (-18.1 to 7.5) | |  |
| MCP III | **-9.4 (-17.0 to -1.8)** | | | -3.1 (-14.4 to 8.2) | | -6.0 (-19.9 to 7.9) | |  |
| MCP IV | **-16.5 (-25.6 to -7.4)** | | | -9.8 (-24.0 to 4.4) | | -6.9 (-23.5 to 9.6) | |  |
| MCP V | -9.6 (-19.3 to 0.1) | | | -15.0 (-32.0 to 2.0) | | 5.8 (-12.1 to 23.7) | |  |
| PIP I | **-8.9 (-17.6 to -0.1)** | | | -8.9 (21.6 to 3.8) | | -7.3 (-23.8 to 9.2) | |  |
| PIP II | **-10.5 (-18.7 to -2.3)** | | | -8.9 (-20.5 to 2.6) | | -11.1 (-26.2 to 3.9) | |  |
| PIP III | **-11.2 (-19.0 to -3.4)** | | | -6.4 (-17.9 to 5.0) | | -6.4 (-20.3 to 7.6) | |  |
| PIP IV | -6.0 (-13.7 to 1.8) | | | -10.3 (-25.0 to 4.3) | | -7.2 (-23.7 to 9.3) | |  |
| PIP V | **-10.0 (-19.0 to -1.0)** | | | **-22.1 (-36.5 to -7.6)** | | -9.8 (-24.8 to 5.3) | |  |
| Left hand | | | | | | | |  |
| MCP I | -5.9 (-12.8 to 1.1) | | | -6.2 (-14.9 to 2.6) | | **-12.7 (-25.0 to -0.4)** | |  |
| MCP II | **-7.1 (-14.1 to -0)** | | | -7.0 (-16.7 to 2.6) | | **-16.0 (-28.7 to -3.4)** | |  |
| MCP III | -4.7 (-12.2 to 2.8) | | | -6.1 (-17.2 to 5.1) | | **-15.5 (-29.0 to -2.0)** | |  |
| MCP IV | **-10.2 (-19.2 to -1.2)** | | | -8.0 (-23.0 to 6.9) | | -15.7 (-36.2 to 4.8) | |  |
| MCP V | -6.4 (-15.3 to 2.4) | | | -10.2 (-25.4 to 4.9) | | -3.1 (-21.6 to 15.4) | |  |
| PIP I | **-9.8 (-18.4 to -1.2)** | | | -10.5 (-23.3 to 2.3) | | **-17.5 (-34.2 to -0.9)** | |  |
| PIP II | **-8.8 (-16.3 to -1.3)** | | | -8.4 (-21.3 to 4.5) | | -15.4 (-32.2 to 1.4) | |  |
| PIP III | **-11.1 (-18.3 to -3.8)** | | | **-10.6 (-20.8 to -0.3)** | | -12.0 (-27.6 to 3.6) | |  |
| PIP IV | **-8.2 (-15.8 to -0.5)** | | | -2.6 (-14.5 to 9.2) | | -6.4 (-22.7 to 9.8) | |  |
| PIP V | **-8.0 (-15.6 to -0.4)** | | | -6.1 (-19.2 to 7.0) | | **-16.7 (-32.5 to -0.9)** | |  |
| **β (95% confidence interval). MCP = Metacarpophalangeal joint; PIP = Proximal interphalangeal joint. | | | | | | | |  |
|  | | | | | | | | |
| Supplementary Table 9. RF/anti-CCP positive patients with RA  Relation over time between involvement of individual MCP and PIP joint  and grip force# | | | | | | |  |  |
| Synovitis | | **Right hand*** | | | **Left hand*** | |  |  |
| MCP I | | -3.9 (-8.1 to 0.2) | | | -3.0 (-6.6 to 0.5) | |  |  |
| MCP II | | -3.0 (-7.0 to 1.0) | | | -2.2 (-6.0 to 1.6) | |  |  |
| MCP III | | -2.6 (-6.4 to 1.2) | | | -1.6 (-4.8 to 1.7) | |  |  |
| MCP IV | | **-5.3 (-10.0 to -0.6)** | | | -3.3 (-8.7 to 2.1) | |  |  |
| MCP V | | -2.7 (-8.3 to 2.9) | | | **-4.8 (-9.4 to -0.3)** | |  |  |
| PIP I | | 2.2 (-3.7 to 8.1) | | | -1.9 (-6.9 to 3.0) | |  |  |
| PIP II | | -0.4 (-4.6 to 3.7) | | | -1.3 (-6.2 to 3.6) | |  |  |
| PIP III | | 1.0 (-2.8 to 4.8) | | | 0.1 (-4.8 to 5.1) | |  |  |
| PIP IV | | -0.6 (-5.5 to 4.4) | | | -0.8 (-5.5 to 3.9) | |  |  |
| PIP V | | -5.1 (-11.1 to 0.8) | | | -0.8 (-7.2 to 5.7) | |  |  |
| Tenderness Right hand* Left hand* | | | | | | |  |  |
| MCP I | | -3.3 (-8.2 to 1.6) | | | -0.1 (-4.7 to 4.6) | |  |  |
| MCP II | | **-4.3 (-8.5 to -0.1)** | | | -2.1 (-6.5 to 2.3) | |  |  |
| MCP III | | **-4.9 (-9.6 to -0.1)** | | | -0.1 (-4.5 to 4.4) | |  |  |
| MCP IV | | **-9.8 (-14.6 to -5.1)** | | | **-6.5 (-11.8 to -1.1)** | |  |  |
| MCP V | | -2.5 (-9.1 to 4.1) | | | -3.1 (-9.5 to 3.3) | |  |  |
| PIP I | | 0.4 (-6.4 to 7.2) | | | -3.4 (-9.4 to 2.6) | |  |  |
| PIP II | | -5.0 (-10.1 to 0.1) | | | -3.4 (-9.9 to 3.1) | |  |  |
| PIP III | | **-6.0 (-11.1 to -0.9)** | | | -4.7 (-9.6 to 0.2) | |  |  |
| PIP IV | | -4.2 (-10.6 to 2.2) | | | -1.0 (-7.7 to 5.6) | |  |  |
| PIP V | | **-6.5 (-12.8 to -0.2)** | | | -3.7 (-9.9 to 2.4) | |  |  |
| # (% of expected). Generalized estimating equations  *β (95 % CI), adjusted for wrist tenderness, erythrocyte sedimentation rate and pain (visual analogue scale); MCP = Metacarpophalangeal joint; PIP = Proximal interphalangeal joint. | | | | | | |  |  |

| Supplementary Table 10. RF- and anti-CCP negative patients with RA  Relation over time between involvement of individual MCP and PIP joint  and grip force# | | |
| --- | --- | --- |
| Synovitis | **Right hand*** | **Left hand*** |
| MCP I | **-7.9 (-12.7 to -3.1)** | **-4.3 (-8.4 to -0.2)** |
| MCP II | 0.8 (-4.1 to 5.7) | -1.0 (-5.1 to 3.1) |
| MCP III | 3.3 (-2.8 to 9.4) | 2.9 (-2.1 to 7.9) |
| MCP IV | -2.8 (-12.8 to 7.2) | **-15.1 (-23.8 to -6.4)** |
| MCP V | -5.8 (-13.1 to 1.5) | **-11.1 (-22.0 to -0.1)** |
| PIP I | -4.8 (-15.0 to 5.4) | -4.6 (-13.2 to 4.0) |
| PIP II | 1.5 (-5.5 to 8.6) | -3.0 (-8.9 to 2.9) |
| PIP III | -0.6 (-6.7 to 5.6) | -2.5 (-8.2 to 3.2) |
| PIP IV | -0.4 (-7.8 to 6.9) | 3.3 (-5.6 to 12.2) |
| PIP V | 3.6 (-6.1 to 13.3) | 6.9 (-4.1 to 17.9) |
| Tenderness | **Right hand*** | **Left hand*** |
| MCP I | **-10.2 (-15.2 to -5.3)** | -2.7 (-7.2 to 1.8) |
| MCP II | -2.9 (-10.0 to 4.2) | -1.5 (-6.1 to 3.2) |
| MCP III | 1.9 (-5.6 to 9.5) | -0.3 (-5.9 to 5.3) |
| MCP IV | **-5.8 (-11.4 to -0.2)** | -5.6 (-12.9 to 1.8) |
| MCP V | -5.0 (-10.9 to 0.8) | 0.3 (-7.9 to 8.5) |
| PIP I | -5.2 (-11.5 to 1.0) | **-7.5 (-14.6 to -0.3)** |
| PIP II | -2.7 (-10.0 to 4.8) | -1.1 (-6.1 to 3.9) |
| PIP III | 0.1 (-7.6 to 7.8) | -2.4 (-8.1 to 3.3) |
| PIP IV | 5.7 (-3.8 to 15.2) | 3.1 (-8.6 to 14.7) |
| PIP V | 3.5 (-3.9 to 10.9) | 5.1 (-7.0 to 17.3) |
| # (% of expected). Generalized estimating equations  *β (95 % CI), adjusted for wrist tenderness, erythrocyte sedimentation rate and pain (visual analogue scale); MCP = Metacarpophalangeal joint; PIP = Proximal interphalangeal joint. | | |
